# Adherence to and factors associated with the adoption of healthy and sustainable diets in young Australian adults: a cross-sectional study

**DOI:** 10.3389/fnut.2026.1862059

**Published:** 2026-07-22

**Authors:** Bill Tiger Lam, Ewa A. Szymlek-Gay, Christel Larsson, Claire Margerison

**Affiliations:** 1 Deakin Institute for Physical Activity and Nutrition, Deakin University, Geelong, VIC, Australia; 2 Department of Food and Nutrition, and Sport Science, University of Gothenburg, Gothenburg, Sweden

**Keywords:** cross-sectional, dietary adherence, nutrition, sustainable diets, young adults

## Abstract

**Introduction:**

Transitioning toward healthy and sustainable diets is needed to address health and environmental challenges. Young Australian adults have poor diet quality and are thus a pivotal age group for healthy and sustainable dietary intervention. As such, the aim of the study was to examine the adherence and factors influencing adoption of healthy and sustainable diets in young Australian adults.

**Methods:**

Young adults (aged 18–25 years) living in Australia completed an online survey (*n* = 297) which assessed their knowledge, attitudes, barriers and enablers toward healthy and sustainable diets. 107 of the 297 participants completed up to four Automated Self-Administered 24-h diet recalls. Diet recall data were used to calculate adherence to healthy and sustainable diets using a modified version of the World Index for Sustainability and Health (mod-WISH) score. Associations between demographic factors and knowledge, attitudes, barriers, enablers, and mod-WISH score were assessed using univariable and multivariable linear and logistic regression.

**Results:**

Mean mod-WISH score was 49.3 ± 14.5, out of a total possible score of 130. From the multivariable models, those following a plant-based diet had higher mod-WISH score compared to those following a non-plant-based diet (*p* = 0.024). Most participants were not aware of healthy and sustainable diet guidelines, and did not perceive these diets as convenient, affordable or accessible. Preferences and perceived health benefits of meat and dairy were identified as key barriers to following these diets. Compared to females, males were less likely to report that understanding animal welfare concerns motivated them to adopt these diets (*p* = 0.008) and were more likely to report that their preference for meat was a barrier to following these diets (*p* = 0.016). Moreover, full-time employed young adults were less likely to perceive healthy and sustainable diets as convenient (*p* = 0.019), while those who were part-time/casual employed were more likely to agree that having greater access to healthy and sustainable food environments encouraged them toward these diets (*p* = 0.029), compared to unemployed young adults.

**Conclusion:**

Findings from this study emphasize the need to target young Australian adults in healthy and sustainable dietary interventions. Furthermore, results may be useful in the planning and development of such interventions.

## 1 Introduction

Current food systems are major contributors to the double burden of malnutrition and environmental degradation (1). Inadequate food supply in low-income countries leads to energy and nutrient deficiencies, while the over-supply and pervasiveness of ultra-processed foods in middle- and high-income nations increases rates of chronic disease (2, 3). Additionally, food systems are substantial drivers of climate change, land degradation, biodiversity loss, deforestation and water pollution (1, 4). Transforming food production and consumer demand for healthier and more sustainable diets is needed to address these global concerns.

Healthy and sustainable diets, as defined by the Food and Agriculture Organization of the United Nations (FAO), are dietary patterns that promote health and wellbeing, while minimizing negative environmental and social impacts. These diets are accessible, affordable, safe, equitable, culturally acceptable and have a low environmental footprint (5). These diets have a central focus on wholegrains, legumes, nuts, fruits and vegetables, while eggs, dairy, poultry, fish, red meat and ultra-processed foods are moderated (1).

Young adults are a pivotal age group in encouraging demand for healthy and sustainable diets as they are deeply concerned and are aware of the effects of climate change. They are also motivated to pressure governments and businesses to act on the climate crisis (6, 7). Despite this, evidence shows that they lack awareness of how food systems contribute to environmental degradation (7). Young Australian adults also have the poorest quality diets of all adult age groups (8), which may be contributing to their increasing rates of poor mental health (9) and bowel cancer (10). Promoting a diet that aligns with young adults’ pro-environmental values, may lead to improvements in their diet quality and health outcomes, potentially reducing their risk of chronic disease later in life (11).

Before designing targeted interventions to improve adherence to healthy and sustainable diets in young adults, it is vital to gain insight into their current dietary consumption patterns, and the factors that influence their intake. Assessing knowledge, attitudes and behaviors toward healthy eating can inform the design and goals of healthy eating interventions (12–14). Although previous studies among adult populations have investigated knowledge and attitudes toward healthy and sustainable diets (15–17), no identified studies relate this to dietary behaviors (18). There is currently limited information on young adults’ adherence to healthy and sustainable diets using validated dietary assessment and index tools (19). Additionally, little evidence exists that explores the associations of demographic characteristics with knowledge, attitudes, barriers and enablers (17).

As such, the aim of the present study was to investigate the adherence to and factors influencing adoption of healthy and sustainable diets and associations with demographic variables. Specific outcomes that were explored included adherence and knowledge of healthy and sustainable diets, and attitudes, barriers, enablers to adopting these diets.

## 2 Methods

### 2.1 Study design

This cross-sectional study employed an online survey and up to four non-consecutive 24-h dietary recalls to collect healthy and sustainable diet data from young adults aged 18–25 years old across Australia. Data collected included dietary intakes, general nutrition knowledge, and knowledge, attitudes, barriers and enablers toward healthy and sustainable diets. Recruitment and data collection were conducted between February 2024 and October 2024. Reporting of the study follows the Strengthening the Reporting of Observational Studies in Epidemiology – Nutritional Epidemiology (STROBE-nut) checklist (Supplementary material 1) (20).

### 2.2 Participants

The study aimed to recruit a minimum of 104 participants who completed both the survey and at least one 24-h dietary recall. Sample size calculations were informed by an unpublished 2022 pilot study of young Australian adults (aged 18–24 years), which assessed Australian Dietary Guidelines knowledge scores (21) (possible score range: 0–19) and diet type (i.e., plant-based vs. non-plant-based). In the pilot, mean knowledge scores were 15.4 (SD 2.2) for those following a plant-based diet and 14.1 (SD 2.5) for those following a non-plant-based diet. These scores were used to estimate the sample size required to detect differences in nutrition knowledge scores between low and high adherence to a healthy and sustainable diet in the current study. A Welch’s t-test with an *α* = 0.05 and power = 0.8, was used for this calculation. Participants were eligible if they were aged between 18–25 years and were living in Australia. Individuals who were pregnant or breastfeeding or had significantly changed their diet in the previous 6 months were excluded from participation.

### 2.3 Recruitment and ethics

Recruitment involved social media posts on the university’s institution page, and physical flyers posted in Australian university public spaces. The posts and flyers included links to the landing page of the survey. On this page, prospective participants were asked to read the Plain Language Statement and confirm their consent by clicking ‘Yes, I consent’, before starting the survey. The Plain Language Statement included details about the study, including its purpose (to explore young Australian adults’ knowledge, attitudes and behaviors relating to diet, and their dietary intakes). After completion of the survey, they were asked for their consent to provide their dietary data. Participants had a chance to win one of ten A$50 shopping vouchers if they completed the survey and at least two dietary recalls. Participants who completed the survey and four dietary recalls, were granted an additional entry into the prize draw.

Ethics approval for the study was granted by the Faculty of Health Human Ethics Advisory Group at Deakin University (HEAG-H 147_2023).

### 2.4 Data collection

#### 2.4.1 Demographics and characteristics

Participants’ demographics and characteristics were collected via the online survey and included age, gender, education level, main language spoken at home, living arrangements, employment/student status, and current diet. Education level (below university qualification vs. university qualified), language spoken at home (English vs. other languages), living arrangements (living with family vs. living alone or with partner/housemates) and current diet (plant-based vs. non-plant-based) were collapsed into two groups. Plant-based dieters included participants who reported consuming a diet that involved any restriction of animal product intake, i.e., flexitarian, no red meat, pescatarian, vegetarian and vegan. This does not include restriction due to allergies or intolerances. Non-plant-based dieters included participants who consumed a non-restricted diet, i.e., omnivore or consumed other diets, i.e., keto.

#### 2.4.2 Knowledge, attitudes, enablers, and barriers

The online survey (Qualtrics) aimed to assess participants’ knowledge, attitudes, barriers and enablers toward healthy and sustainable diets. The survey was reviewed for content validity by nutrition researchers (*n* = 2) and was pilot-tested within a small sample of young Australian adults (*n* = 3) for construct validity. Minor edits were made for clarity, and some questions were removed to reduce time burden on participants.

The general nutrition knowledge questionnaire (possible score range: 0–20) was adapted from a validated tool (22), while the healthy and sustainable diet knowledge questionnaire (possible score range: 0–13) was based on the EAT-Lancet Planetary Health Diet food group recommendations (1). Questions for attitudes, barriers and enablers toward healthy and sustainable diets were adapted from previous studies investigating plant-based diets (15, 23, 24). Participants’ agreement toward fourteen attitude, seven enabler and five barrier statements were assessed using 4-point Likert scales (strongly disagree to strongly agree). Attitudes statements started with “Healthy and sustainable diets are…,” followed by the attitudes, e.g., “accessible” and “easy to follow.” The barrier and enabler statements started with the prompt “How important are the following factors in encouraging/hindering you to adopt a healthy and sustainable diet?,” followed by the barrier or enabler, e.g., “I like to eat meat.” All attitudes, enablers and barriers were collapsed into a binary variable, i.e., disagree vs. agree, to conduct analysis.

#### 2.4.3 Dietary intakes

Following completion of the survey, participants were asked to complete four non-consecutive 24-h dietary recalls (three weekdays and one weekend day) over a two-week period using the Australian online Automated Self-Administered 24-h (ASA24-Australia) dietary assessment tool. This tool was based on the United States Department of Agriculture’s Automated Multiple-Pass Method which has been validated to estimate total energy and protein intakes (25). The ASA24-Australia is adapted to the Australian context and uses the Australian Food, Supplement and Nutrient Database (AUSNUT) 2011-13 files to guide food inclusions, portion sizes and nutrient calculations. Although supplement intake was recorded in the ASA24-Australia tool, this is not reported as micronutrient intake was not investigated in this study.

Each food item in the dietary data was disaggregated into the World Index for Sustainability and Health (WISH) 13 food components (26). A list of the food components, along with the foods included and excluded in each component, can be found in Supplementary material 2. For mixed foods, the AUSNUT recipe files were used to calculate gram amounts for each relevant food group. If no recipe data existed for mixed foods, the percentage of each ingredient was extracted from nutrition information panels. These were collected from Australian supermarket or recipe websites. Once the grams of each food group were calculated for each listed food in the dietary data, usual food group intakes in grams per day were estimated using the Multiple Source Method (MSM) (27). Habitual consumers for dairy, poultry, seafood, eggs and red meat were adjusted for in the MSM model. Consumers of these food components were identified using data reported in the survey. For example, if a participant reported following a lacto-ovo vegetarian diet, they would be non-consumers of poultry, seafood and red meat. Further, MSM models were adjusted according to age, gender, day of recall (i.e., weekday vs. weekend day), and energy intake. Usual intakes in grams per day were used to assess adherence to a healthy and sustainable diet which was measured using a modified version of the WISH tool (mod-WISH) (26), which takes into account the new 2025 EAT-Lancet Planetary Health Diet guidelines (28).

The mod-WISH score for each of the 13 food components was scored on a continuous range between 0–10, with the exception of saturated oils and added sugar which was a binary score of 0 or 10. A score of 0 was generated if intakes were either below the lower recommended intake or above the upper recommended intake, with the exception of wholegrains, fruits, vegetables and legumes where there were no restrictions for the upper range. The maximum score of 10 was achieved if intakes were within recommended ranges. Total mod-WISH score (0–130) was calculated by aggregating the score of each food component. Higher scores indicate higher adherence to the 2025 EAT-Lancet Planetary Health Diet recommendations.

### 2.5 Data analysis

Participant survey data were included if they completed any section of the survey, in addition to providing demographic data. Participant dietary data were included if at least one 24-h recall was completed. Mis-reporters were identified if their mean energy intake was three standard deviations above or below the group mean. Means, standard deviations, frequencies, percentages, median, and interquartile range were reported for knowledge scores, attitudes, mod-WISH scores, and intake of food groups. Univariable (unadjusted) linear regression was conducted to assess associations. Independent variables included participant characteristics (i.e., demographics and current diet type), while dependent variables included general nutrition score, healthy and sustainable diet knowledge score and mod-WISH score. Model assumptions, including homoscedasticity and normality of residuals, were assessed using residual plots. Univariable (unadjusted) binary logistic regression was performed to identify associations between participant characteristics (independent variables) and the dependent variables, which included agreement with the 14 attitude, five barrier, and seven enabler statements regarding healthy and sustainable diets. Minimum sample sizes (>15) were checked using counts to assess model assumptions. Firth’s penalized logistic regression was used if there was 100% agreement within demographic groups. Analysis was not conducted for one outcome variable, i.e., ‘health’ attitude, as there were fewer than 15 participants in one group. Multivariable models were then fitted for outcome variables where at least two independent variables were associated in the univariable models. A conservative approach was used, whereby independent variables with *p* < 0.1 in the univariable models were included in the multivariable models. Data were analyzed using Stata (Version 18). *p* < 0.05 denoted statistical significance.

## 3 Results

### 3.1 Participants

**Figure 1:**
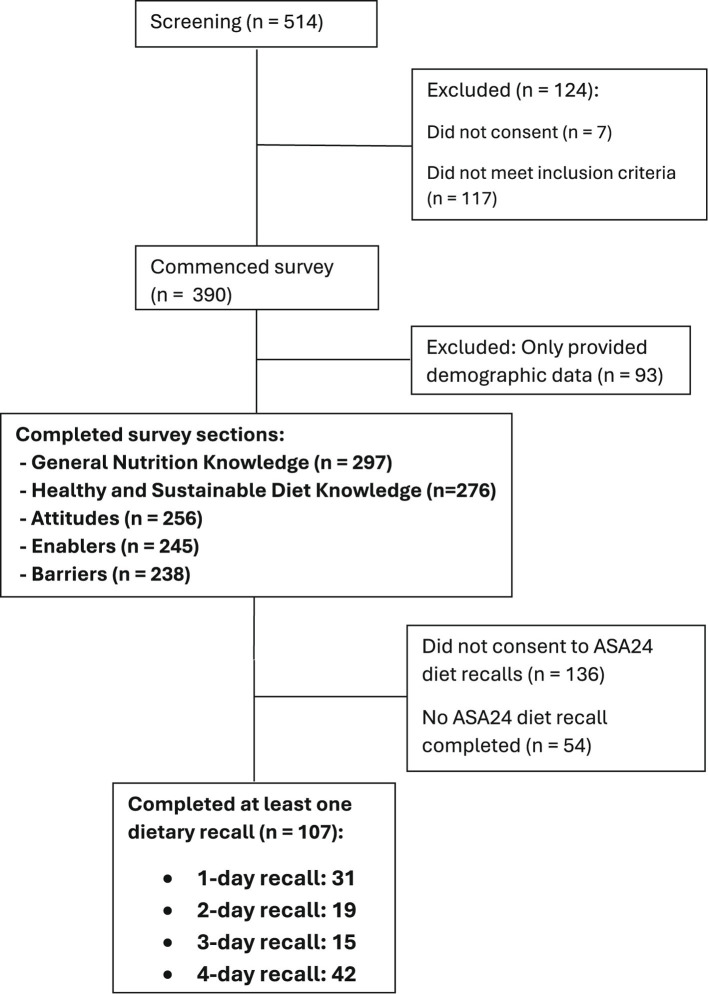
Participant flow diagram.

A total of 514 individuals opened the online survey and commenced the consent and screening process (Figure 1). Of these, 390 consented and met the inclusion criteria. Two hundred and ninety-seven participants completed at least one section of the survey and were included in the analysis. Two hundred and thirty-eight completed all sections of the survey. One hundred and seven completed at least one dietary recall. No participants were excluded due to misreporting.

The mean (±SD) age of all participants (*n* = 297) was 21.0 ± 2.4 years. Among all participants, 68% identified as female, 79% had not completed a university-level qualification, 64% were employed, 85% were current students, and 69% followed a non-plant-based diet (Table 1). The mean energy intake among those who completed at least one dietary recall (*n* = 107) was 7,260 ± 1989 kJ/d (Supplementary material 3).

**Table 1:** Demographic and dietary characteristics of all participants (*n* = 297) and those who provided dietary data (*n* = 107).

Characteristic	All participants (*n* = 297) *n* (%)	Provided dietary data (*n* = 107) *n* (%)
Gender		
Female	202 (68%)	73 (69%)
Male	83 (28%)	28 (26%)
Gender diverse or not specified	12 (4%)	6 (5%)
Education level		
Below university level^1^	235 (79%)	71 (66%)
University qualification	62 (21%)	36 (34%)
Main language spoken at home		
English	208 (70%)	77 (72%)
Other^2^	89 (30%)	30 (28%)
Living arrangements		
Living alone or with partner/housemates	158 (53%)	58 (54%)
Living with parents and/or other family members	139 (47%)	49 (46%)
Employment status		
Employed part-time/casual	162 (55%)	54 (50%)
Unemployed	107 (36%)	36 (34%)
Employed full-time	28 (9%)	17 (16%)
Student status		
Studying full-time	232 (79%)	78 (73%)
Not studying	46 (15%)	24 (22%)
Studying part-time	19 (6%)	5 (5%)
Current diet		
Omnivore (no restrictions)	200 (67%)	73 (68%)
Lacto-ovo vegetarian^3^	34 (11%)	7 (7%)
Vegan	24 (8%)	9 (8%)
No red meat	20 (7%)	7 (7%)
Flexitarian	19 (6%)	8 (7%)
Keto	5 (2%)	1 (1%)
Pescatarian	4 (1%)	2 (2%)

^1^Qualifications below university level, e.g., secondary school or vocational. Most participants (*n* = 202) were currently enrolled in university. ^2^Includes Mandarin, Cantonese, Vietnamese, Indonesian, Hindi, Malayalam, Gujarati, Bengali, Sinhalese, Tamil, Japanese, German, Korean, Sinhalese. ^3^Vegetarian diet that excludes meat, poultry, fish but includes dairy products and eggs.

### 3.2 Knowledge of nutrition and healthy and sustainable diets

#### 3.2.1 General nutrition knowledge scores

Out of a maximum possible score of 20 for general nutrition knowledge, the mean score of all participants (*n* = 297) was 13.4 ± 3.7 and ranged from 2 to 20. Four participants scored the maximum score of 20. For 16 out of the 20 individual general nutrition knowledge questions, more than half of participants answered correctly (Supplementary material 4). Less than half of participants correctly answered the four questions: ‘Skim milk contains fewer minerals than full-fat milk’ (30%), ‘Brown sugar is much healthier than white sugar’ (42%), ‘Bacon contains more calories than ham’ (47%), ‘A sandwich with mozzarella contains as many calories as the same sandwich with cheddar cheese’ (49%).

In the univariable models, females had higher scores than males (*p* = 0.009), those who spoke English in their household had higher scores than those who did not (*p* = 0.020) and employed individuals had higher scores compared to unemployed individuals (overall *p* = 0.018; Table 2). However, there were no significant differences between participant characteristics and general nutrition knowledge scores in the multivariable model (all *p* > 0.05).

**Table 2:** Associations between participant characteristics and general nutrition knowledge scores (*n* = 297).

Characteristics	General nutrition knowledge score^1^ (Mean (SD))	Univariable beta (95% CI)	Univariable *p*-value^2^	Multivariable beta (95% CI)	Multivariable *p*-value^3^
Age		0.0 (−0.2, 0.2)	0.758		
Gender			0.033		0.065
Female (reference)	13.8 (3.6)				
Male	12.5 (4.1)	−1.3 (−2.2, −0.3)	0.009	−1.1 (−2.1, −0.2)	0.019
Gender diverse or not specified	13.6 (2.4)	−0.2 (−2.3, 2.0)	0.877	−0.2 (−2.4, 1.9)	0.845
Education level					
Below university level^4^ (reference)	13.2 (3.7)				
University qualification	14.2 (3.6)	1.0 (−0.0, 2.1)	0.057	1.0 (−0.3, 2.3)	0.129
Main language spoken at home					
English (reference)	13.7 (3.6)				
Other^5^	12.6 (3.8)	−1.1 (−2.0, −0.2)	0.020	−0.9 (−1.8, 0.1)	0.075
Living arrangements					
Living alone or with partner/housemates (reference)	13.2 (3.7)				
Living with parents and/or other family members	13.6 (3.8)	0.4 (−0.4, 1.3)	0.299		
Employment status			0.018		0.248
Unemployed (reference)	12.7 (3.8)				
Employed part-time/casual	13.7 (3.6)	1.0 (0.1, 1.9)	0.033	0.6 (−0.3, 1.6)	0.184
Employed full-time	14.6 (3.3)	2.0 (0.4, 3.5)	0.012	1.4 (−0.5, 3.3)	0.157
Student status			0.088		0.143
Not studying (reference)	14.2 (3.5)				
Studying part-time	12 (4.3)	−2.2 (−4.2, −0.2)	0.030	−1.4 (−3.4, 0.6)	0.182
Studying full-time	13.3 (3.7)	−0.8 (−2.0, 0.3)	0.157	0.4 (−1.1, 1.8)	0.616
Current diet^6^					
Non plant-based (reference)	13.4 (3.5)				
Plant-based	13.4 (4.2)	−0.0 (−0.9, 0.9)	0.983		

^1^Possible score range is 0–20. ^2^Derived from linear regression (univariable analysis). ^3^Derived from linear regression (multivariable analysis) where gender, education level, language spoken at home, employment status and student status were used as covariates. Independent variables that were not associated with the outcome in the univariable models were not included in the multivariable analysis. ^4^Qualifications below university level, e.g., secondary school or vocational. Most participants (*n* = 202) were currently enrolled in university. ^5^Includes Mandarin, Cantonese, Vietnamese, Indonesian, Hindi, Malayalam, Gujarati, Bengali, Sinhalese, Tamil, Japanese, German, Korean, Sinhalese. ^6^Plant-based dieters were participants who reported following a diet that restricted animal product intake, i.e., flexitarian, no red meat, pescatarian, vegetarian and vegan. This definition excludes restrictions due to allergies or intolerances. Non-plant-based dieters were participants who consumed an unrestricted diet, i.e., omnivore, or other diets, i.e., keto (*n* = 5).

#### 3.2.2 Healthy and sustainable diet knowledge scores

For healthy and sustainable diet knowledge, participants (*n* = 276) scored a mean of 7.6 ± 2.2 out of a possible of 13 points (range: 0 to 13). Three participants achieved the maximum score of 13. For 6 out of 13 individual healthy and sustainable diet knowledge questions, more than half the participants answered correctly (Supplementary material 5). Less than half correctly categorized the following food groups as either encouraged, optional or discouraged in a healthy and sustainable diet: refined grains (24%), red meat (44%), poultry (27%), fish (27%), dairy (42%), eggs (29%), and soy foods (37%) (Supplementary material 5).

In the univariable models, females had higher scores than males (*p* = 0.034), participants with a university qualification had higher scores than those without (*p* = 0.042), part-time/casually employed individuals had higher scores than those who were unemployed (*p* = 0.027), and participants following a plant-based diet had higher scores compared to those not following a plant-based diet (*p* < 0.001) (Table 3). Participants with a higher general nutrition knowledge score also had a higher healthy and sustainable diet knowledge score (*p* < 0.001). In the multivariable model, only higher general nutrition knowledge score (*p* < 0.001) and following a plant-based diet (*p* < 0.001) were positively associated with healthy and sustainable diet knowledge scores.

**Table 3:** Associations between participant characteristics and healthy and sustainable diet knowledge scores (*n* = 276).

Characteristics	Healthy and sustainable diet score^1^ (Mean (SD))	Univariable beta (95% CI)	Univariable *p*-value^2^	Multivariable beta (95% CI)	Multivariable *p*-value^3^
Age		0.1 (−0.0, 0.2)	0.091	0.1 (−0.1, 0.2)	0.315
General Nutrition Knowledge Score		0.2 (0.1, 0.3)	<0.001	0.2 (0.1, 0.2)	<0.001
Gender			0.029		0.159
Female (reference)	7.7 (2.2)				
Male	7.1 (2.1)	−0.6 (−1.2, −0.0)	0.034	−0.3 (−0.9, 0.2)	0.227
Gender diverse or not specified	8.6 (2.4)	0.8 (−0.4, 2.1)	0.187	0.8 (−0.4, 1.9)	0.184
Education level					
Below university level^4^ (reference)	7.5 (2.1)				
University qualification	8.1 (2.3)	0.6 (0.0, 1.2)	0.042	0.2 (−0.6, 1.0)	0.595
Main language spoken at home					
English (reference)	7.7 (2.0)				
Other^5^	7.4 (2.4)	−0.3 (−0.9, 0.2)	0.244		
Living arrangements					
Living alone or with partner/housemates (reference)	7.8 (2.3)				
Living with parents and/or other family members	7.4 (2.0)	−0.3 (−0.9, 0.2)	0.175		
Employment status			0.047		0.444
Unemployed (reference)	7.2 (2.1)				
Employed part-time/casual	7.8 (2.2)	0.6 (0.1, 1.2)	0.027	0.3 (−0.2, 0.8)	0.206
Employed full-time	8.0 (1.9)	0.8 (−0.1, 1.9)	0.070	0.3 (−0.7, 1.2)	0.588
Student status			0.313		
Not studying (reference)	8.0 (2.1)				
Studying part-time	7.7 (2.1)	−0.4 (−1.6, 0.8)	0.530		
Studying full-time	7.5 (2.2)	−0.5 (−1.2, 0.2)	0.129		
Current diet^6^					
Non plant-based (reference)	7.2 (2.1)				
Plant-based	8.5 (2.1)	1.3 (0.8, 1.8)	<0.001	1.2 (0.7, 1.7)	<0.001

^1^Possible score range is 0–13. ^2^Derived from linear regression (univariable analysis). ^3^Derived from linear regression (multivariable analysis) where age, general nutrition knowledge score, gender, education level and employment status were used as covariates. Independent variables that were not associated with the outcome in the univariable models were not included in the multivariable analysis. ^4^Qualifications below university level, e.g., secondary school or vocational. Most participants (*n* = 183) were currently enrolled in university. ^5^Includes Mandarin, Cantonese, Vietnamese, Indonesian, Hindi, Malayalam, Gujarati, Bengali, Sinhalese, Tamil, Japanese, German, Korean, Sinhalese. ^6^Plant-based dieters included participants who reported consuming a diet that involved any sort of restriction of animal product intake, i.e., flexitarian, no red meat, pescatarian, vegetarian and vegan. This does not include restriction due to allergies or intolerances. Non plant-based dieters included participants who consumed a non-restricted diet, i.e., omnivore, or consumed other diets, i.e., keto.

### 3.3 Attitudes toward healthy and sustainable diets

**Figure 2:**
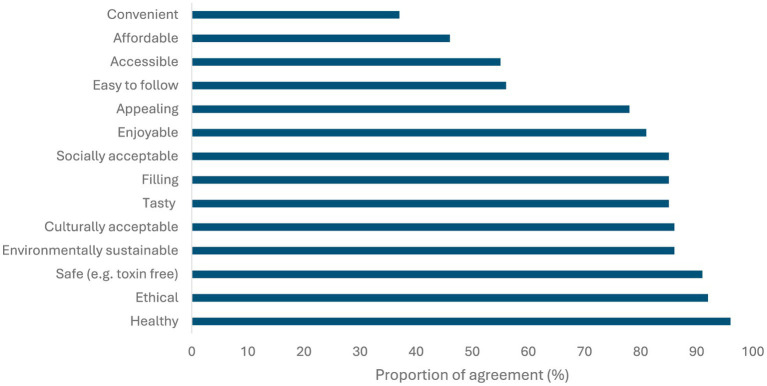
Attitudes toward healthy and sustainable diets^†^ (*n* = 256). ^†^Participants were asked to rate the following on a 4-point Likert scale (from strongly disagree to strongly agree) with the following prompt: Healthy and sustainable diets are [attitude]. They were also provided with the definition of a healthy and sustainable diet in the question: “Healthy and sustainable diets encourage wholefoods such as fruits, vegetables, whole grains and plant proteins. These diets are flexible and allow some eggs, dairy, poultry, fish and red meat. Junk foods and drinks are limited.” Proportion of agreement (%) was calculated by calculating the proportion of those who selected ‘agree” or ‘strongly agree’ on the Likert scales.

Figure 2 presents the proportion of participants (*n* = 256) who selected ‘agree’ or ‘strongly agree’ to the 14 attitude statements. Nearly all (96%) believed that healthy and sustainable diets were healthy. For ten statements, there was over 75% agreement, however, approximately half (56%) of participants agreed that healthy and sustainable diets were easy to follow. A similar proportion agreed they were accessible (55%), whereas 46% agreed they were affordable and 37% agreed they were convenient.

In the univariable models, part-time/casually employed (*p* = 0.032) and full-time employed individuals (*p* = 0.014) were less likely than unemployed individuals to agree that healthy and sustainable diets were convenient (Table 4). However, significant associations only remained for full-time individuals in the multivariable model (*p* = 0.019). Those with a university degree (*p* = 0.029) and who did not speak English at home (*p* = 0.035), were more likely than their counterparts to believe that healthy and sustainable diets were environmentally sustainable in the univariable models. However, there were no significant associations in the multivariable model (all *p* > 0.05) Individuals living with family were more likely to believe healthy and sustainable diets were ethical, compared to those living independently (*p* = 0.049), which was also evident in the multivariable model (*p* = 0.047). In the univariable models, plant-based dieters were more likely to believe that healthy and sustainable diets were affordable (*p* = 0.34), enjoyable (*p* = 0.012) and tasty (*p* = 0.43) compared to those not on a plant-based diet. Compared to females, gender diverse individuals were less likely to believe these diets to be safe for consumption (*p* < 0.001). No associations were identified between other demographic characteristics and attitudes toward healthy and sustainable diets in the univariable and multivariable models.

**Table 4:** Associations between participant characteristics (*n* = 256) and agreement with attitude statements that showed significant correlations^1^.

Characteristics	Healthy and sustainable diets are convenient					Healthy and sustainable diets are environmentally sustainable					Healthy and sustainable diets are ethical				
	*n* (%) who agreed to statement	Univariable OR (95% CI)	Univariable *p*-value^2^	Multivariable OR (95% CI)	Multivariable *p*-value^3^	*n* (%) who agreed to statement	Univariable OR (95% CI)	Univariable *p*-value^2^	Multivariable OR (95% CI)	Multivariable *p*-value^3^	*n* (%) who agreed to statement	Univariable OR (95% CI)	Univariable *p*-value^2^	Multivariable OR (95% CI)	Multivariable *p*-value^3^
Age		1.0 (0.9, 1.1)	0.502				1.2 (1.0, 1.4)	0.062	1.0 (0.8, 1.2)	0.852		0.9 (0.8, 1.1)	0.350		
Gender			0.273					0.781					0.098		0.094
Female (reference)	63 (37%)					149 (87%)					161 (94%)				
Male	30 (42%)	1.2 (0.7, 2.2)	0.460			60 (83%)	0.8 (0.4, 1.6)	0.504			66 (92%)	0.8 (0.3, 2.1)	0.589	0.8 (0.3, 2.4)	0.732
Gender diverse or not specified	2 (17%)	0.3 (0.1, 1.6)	0.179			10 (83%)	0.8 (0.2, 3.7)	0.748			8 (75%)	0.2 (0.0, 0.8)	0.031	0.2 (0.0, 0.9)	0.030
Education level															
Below university level^4^ (reference)	75 (38%)					163 (83%)					181 (92%)				
University qualification	20 (34%)	0.8 (0.5, 1.5)	0.561			56 (95%)	3.9 (1.2, 13.2)	0.029	3.2 (0.8, 13.0)	0.100	55 (93%)	1.2 (0.4, 3.8)	0.736		
Main language spoken at home															
English (reference)	62 (34%)					151 (83%)					168 (92%)				
Other5	33 (45%)	1.6 (0.9, 2.8)	0.092	1.4 (08, 2.5)	0.278	68 (93%)	2.9 (1.1, 7.7)	0.035	2.5 (0.9, 6.8)	0.076	68 (93%)	1.2 (0.4, 3.5)	0.717		
Living arrangements															
Living alone or with partner/housemates (reference)	50 (37%)					117 (86%)					121 (89%)				
Living with parents and/or other family members	45 (38%)	1.0 (0.6, 1.7)	0.903			102 (85%)	0.9 (0.5, 1.8)	0.815			115 (96%)	2.9 (1.0, 8.1)	0.049	2.9 (1.0, 8.4)	0.047
Employment status			0.016		0.034			0.390					0.630		
Unemployed (reference)	45 (47%)					83 (87%)					86 (91%)				
Employed part-time/casual	45 (33%)	0.6 (0.3, 1.0)	0.032	0.6 (0.3, 1.1)	0.075	112 (83%)	0.7 (0.3, 1.5)	0.361			125 (93%)	1.3 (0.5, 3.4)	0.576		
Employed full-time	5 (19%)	0.3 (0.1, 0.8)	0.014	0.3 (0.1, 0.8)	0.019	24 (92%)	1.7 (0.4, 8.3)	0.490			25 (96%)	2.6 (0.3, 21.7)	0.461		
Student status			0.222					0.880					0.690		
Not studying (reference)	11 (26%)					37 (86%)					39 (91%)				
Studying part-time	7 (44%)	2.3 (0.7, 7.5)	0.183			13 (81%)	0.7 (0.2, 3.2)	0.650			14 (88%)	0.7 (0.1, 4.4)	0.719		
Studying full-time	77 (39%)	1.9 (0.9, 3.9)	0.099			169 (86%)	1.0 (0.4, 2.5)	0.965			183 (93%)	1.3 (0.4, 4.3)	0.621		
Current diet^6^															
Non plant-based (reference)	61 (34%)					150 (84%)					163 (91%)				
Plant-based	34 (44%)	1.5 (0.9, 2.6)	0.127			69 (90%)	1.7 (0.7, 3.8)	0.229			73 (95%)	1.8 (0.6, 5.5)	0.312		

Characteristics	Healthy and sustainable diets are affordable					Healthy and sustainable diets are enjoyable			Healthy and sustainable diets are tasty			Healthy and sustainable diets are safe (e.g., toxin free)		
	*n* (%) who agreed to statement	Univariable OR (95% CI)	Univariable *p*-value^2^	Multivariable OR (95% CI)	Multivariable *p*-value^3^	*n* (%) who agreed to statement	Univariable OR (95% CI)	Univariable *p*-value^2^	*n* (%) who agreed to statement	Univariable OR (95% CI)	Univariable *p*-value^2^	*n* (%) who agreed to statement	Univariable OR (95% CI)	Univariable *p*-value^2^
Age		1.1 (1.0, 1.2)	0.053	1.1 (0.9, 1.2)	0.274		1.0 (0.9, 1.1)	0.727		1.0 (0.8, 1.1)	0.747		0.9 (0.7, 1.1)	0.196
Gender			0.763					0.282			0.629			<0.001
Female (reference)	75 (44%)					144 (84%)			148 (86%)			160 (93%)		
Male	36 (50%)	1.3 (0.7, 2.2)	0.361			54 (75%)	0.6 (0.3, 1.1)	0.115	59 (82%)	0.7 (0.4, 1.5)	0.416	66 (92%)	0.8 (0.3, 2.3)	0.712
Gender diverse or not specified	6 (50%)	1.3 (0.4, 4.2)	0.537			10 (83%)	1.0 (0.2, 4.7)	0.972	11 (92%)	1.8 (0.2, 14.5)	0.588	6 (50%)	0.1 (0.0, 0.3)	<0.001
Education level														
Below university level^4^ (reference)	84 (43%)					161 (82%)			170 (86%)			180 (91%)		
University qualification	33 (56%)	1.7 (0.9, 3.1)	0.074	1.5 (0.6, 3.3)	0.379	47 (80%)	0.9 (0.4, 1.8)	0.722	48 (81%)	0.7 (0.3, 1.5)	0.351	52 (88%)	0.7 (0.3, 1.8)	0.456
Main language spoken at home														
English (reference)	78 (43%)					152 (83%)			157 (86%)			164 (90%)		
Other^5^	39 (53%)	1.5 (0.9, 2.7)	0.118			56 (77%)	0.7 (0.3, 1.3)	0.242	61 (84%)	0.8 (0.4, 1.8)	0.651	68 (93%)	1.6 (0.6, 4.4)	0.385
Living arrangements														
Living alone or with partner/housemates (reference)	58 (43%)					106 (78%)			112 (82%)			123 (90%)		
Living with parents and/or other family members	59 (49%)	1.3 (0.8, 2.1)	0.296			102 (85%)	1.6 (0.8, 3.1)	0.151	106 (88%)	1.6 (0.8, 3.3)	0.182	109 (90%)	1.0 (0.5, 2.4)	0.914
Employment status			0.098		0.296			0.551			0.359			0.444
Unemployed (reference)	48 (51%)					75 (79%)			80 (84%)			89 (94%)		
Employed part-time/casual	56 (41%)	0.7 (0.4, 1.2)	0.175	0.7 (0.4, 1.1)	0.137	110 (81%)	1.2 (0.6, 2.3)	0.634	116 (86%)	1.1 (0.5, 2.4)	0.718	120 (89%)	0.5 (0.2, 1.4)	0.220
Employed full-time	13 (50%)	1.0 (0.4, 2.3)	0.962	0.6 (0.2, 1.7)	0.354	23 (88%)	2.0 (0.6, 7.5)	0.281	22 (85%)	1.0 (0.3, 3.4)	0.960	23 (88%)	0.5 (0.1, 2.2)	0.376
Student status			0.127					0.700			0.587			0.511
Not studying (reference)	21 (49%)					36 (84%)			37 (86%)			37 (86%)		
Studying part-time	9 (56%)	1.3 (0.4, 4.3)	0.613			14 (88%)	1.4 (0.3, 7.4)	0.720	14 (88%)	1.1 (0.2, 6.3)	0.885	15 (94%)	2.4 (0.3, 22.0)	0.428
Studying full-time	87 (44%)	0.8 (0.4, 1.6)	0.577			158 (80%)	0.8 (0.3, 1.9)	0.596	167 (85%)	0.9 (0.4, 2.3)	0.832	180 (91%)	1.7 (0.6, 4.6)	0.287
Current diet^6^														
Non plant-based (reference)	74 (41%)					138 (77%)			147 (82%)			161 (90%)		
Plant-based	43 (56%)	1.8 (1.0, 3.1)	0.034	1.8 (1.1, 3.2)	0.032	70 (90%)	3.0 (1.3, 7.0)	0.012	71 (92%)	2.6 (1.0, 6.4)	0.043	71 (92%)	1.3 (0.5, 3.5)	0.570

^1^Participants were asked to rate the attitude statements on a 4-point Likert scale (from strongly disagree to strongly agree). They were also provided with the definition of a healthy and sustainable diet in the question: “Healthy and sustainable diets encourage wholefoods such as fruits, vegetables, whole grains and plant proteins. These diets are flexible and allow some eggs, dairy, poultry, fish and red meat. Junk foods and drinks are limited.” Data were collapsed into a binary variable and % agreement was calculated by combining those who ‘agreed’ or ‘strongly agreed’ to the prompt. ^2^Derived from logistic regression (univariable analysis). ^3^Derived from logistic regression (multivariable analysis) where at least two participant characteristics with *p* < 0.1 in the univariable model were used as covariates. Independent variables that were not associated with the outcome in the univariable models were not included in the multivariable analysis. Multivariable analysis was not conducted for outcomes where there was only one participant characteristic associated. ^4^Qualifications below university level, e.g., secondary school or vocational. Most participants (*n* = 167) were currently enrolled in university.^5^Includes Mandarin, Cantonese, Vietnamese, Indonesian, Hindi, Malayalam, Gujarati, Bengali, Sinhalese, Tamil, Japanese, German, Korean, Sinhalese. ^6^Plant-based dieters included participants who reported consuming a diet that involved any sort of restriction of animal product intake, i.e., flexitarian, no red meat, pescatarian, vegetarian and vegan. This does not include restriction due to allergies or intolerances. Non plant-based dieters included participants who consumed other diets, i.e., keto, or consumed a non-restricted diet, i.e., omnivore.

### 3.4 Enablers to following a healthy and sustainable diet

**Figure 3:**
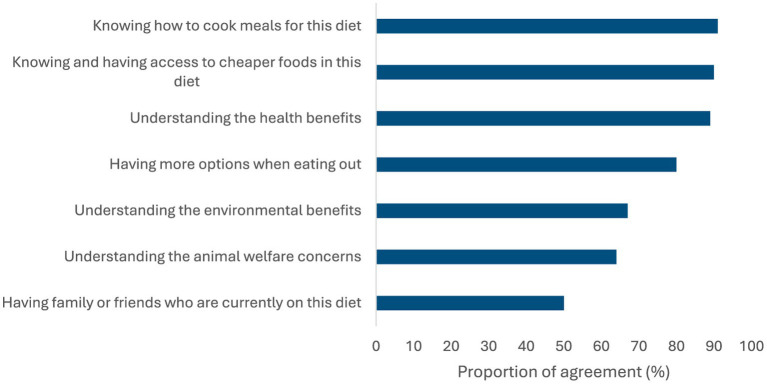
Proportion of participants who agreed to the following enabler statements^†^ in encouraging adoption or maintenance of a healthy and sustainable diet (*n* = 245). ^†^Participants were asked to rate their agreement on a 4-point Likert scale (Strongly disagree to strongly agree) of the seven enablers in encouraging them to adopt or maintain a healthy and sustainable diet. Proportion of agreement was calculated by dividing the number of participants who ‘agreed’ or ‘strongly agreed’ by the total number of participants (*n* = 245).

Figure 3 presents the proportion of participants (*n* = 245) who selected ‘agree’ or ‘strongly agree’ to the seven enabler statements. For all seven enablers, most participants agreed that these would encourage them to adopt or maintain a healthy and sustainable diet.

In the univariable models, males were less likely to agree that ‘understanding the environmental benefits’ (*p* = 0.022) (Table 5) and ‘understanding the animal welfare concerns’ (*p* = 0.006) would encourage them to adopt or maintain a healthy and sustainable diet, compared to females. However, in the multivariable models, this was only significant for ‘understanding the animal welfare concerns’ (*p* = 0.008). Those on a plant-based diet were more likely to agree that ‘understanding the environmental benefits (*p* = 0.006) and ‘understanding the animal welfare concerns’ (*p* < 0.001) would encourage them to adopt or maintain a healthy and sustainable diet, compared to those not following a plant-based diet. This was also evident in the multivariable models (all *p* < 0.05). In the univariable model, employed individuals were also more likely to agree that ‘knowing and having access to cheaper foods in this diet’ would encourage them to adopt or maintain a healthy and sustainable diet, compared to unemployed individuals (*p* = 0.028). No associations were found between other demographic groups and enablers to following a healthy and sustainable diet in either univariable or multivariable models.

**Table 5:** Associations between participant characteristics (*n* = 245) and agreement with enabler statements that showed significant correlations^1^.

Characteristics	Understanding the environmental benefits					Understanding the animal welfare concerns					Knowing and having access to cheaper foods in this diet		
	*n* (%) who agreed to statement	Univariable OR (95% CI)	Univariable *p*-value^2^	Multivariable OR (95% CI)	Multivariable *p*-value^3^	*n* (%) who agreed to statement	Univariable OR (95% CI)	Univariable *p*-value^2^	Multivariable OR (95% CI)	Multivariable *p*-value^3^	*n* (%) who agreed to statement	Univariable OR (95% CI)	Univariable *p*-value^2^
Age		1.0 (0.9, 1.2)	0.406				1.0 (0.9, 1.1)	0.904				1.0 (0.9, 1.3)	0.604
Gender			0.044		0.084			0.021		0.028			0.742
Female (reference)	117 (72%)					114 (70%)					146 (90%)		
Male	41 (57%)	0.5 (0.3, 0.9)	0.022	0.5 (0.3, 0.9)	0.029	37 (51%)	0.4 (0.3, 0.8)	0.006	0.4 (0.2, 0.8)	0.008	64 (89%)	0.9 (0.4, 2.1)	0.726
Gender diverse or not specified	7 (64%)	0.7 (0.2, 2.4)	0.543	0.6 (0.2, 2.2)	0.424	7 (64%)	0.7 (0.2, 2.6)	0.638	0.6 (0.1, 2.2)	0.415	11 (100%)	2.6 (0.1, 46.0)	0.517
Education level													
Below university level^4^ (reference)	124 (66%)					119 (63%)					169 (90%)		
University qualification	41 (72%)	1.3 (0.7, 2.5)	0.400			39 (68%)	1.3 (0.7, 2.4)	0.479			52 (91%)	1.2 (0.4, 3.3)	0.767
Main language spoken at home													
English (reference)	120 (67%)					117 (65%)					160 (89%)		
Other^5^	45 (69%)	1.1 (0.6, 1.9)	0.866			41 (62%)	0.9 (0.5, 1.6)	0.638			61 (92%)	1.4 (0.5, 4.1)	0.480
Living arrangements													
Living alone or with partner/housemates (reference)	89 (67%)					84 (64%)					117 (89%)		
Living with parents and/or other family members	76 (67%)	1.0 (0.6, 1.7)	0.978			74 (65%)	1.1 (0.6, 1.8)	0.763			104 (92%)	1.5 (0.6, 3.5)	0.375
Employment status			0.387					0.610					0.035
Unemployed (reference)	60 (67%)					58 (64%)					75 (83%)		
Employed part-time/casual	84 (65%)	0.9 (0.5, 1.6)	0.812			81 (63%)	0.9 (0.5, 1.6)	0.803			120 (93%)	2.7 (1.1, 6.4)	0.029
Employed full-time	21 (81%)	2.1 (0.7, 6.1)	0.174			19 (73%)	1.5 (0.6, 3.9)	0.414			26 (100%)	0.9 (0.6, 188.2)	0.101
Student status			0.954					0.369					0.414
Not studying (reference)	27 (64%)					27 (64%)					40 (95%)		
Studying part-time	13 (81%)	2.4 (0.6, 9.8)	0.220			13 (81%)	2.4 (0.6, 9.8)	0.220			15 (94%)	0.8 (0.1, 8.9)	0.820
Studying full-time	125 (67%)	1.1 (0.6, 2.3)	0.751			118 (63%)	1.0 (0.5, 1.9)	0.886			166 (89%)	0.4 (0.1, 1.8)	0.222
Current diet^6^													
Non plant-based (reference)	105 (62%)					93 (55%)					154 (91%)		
Plant-based	60 (80%)	2.5 (1.3, 4.7)	0.006	2.5 (1.3, 4.7)	0.007	65 (87%)	5.4 (2.6, 11.2)	<0.001	5.5 (2.6, 11.5)	<0.001	67 (89%)	0.9 (0.4, 2.1)	0.761

^1^Participants were asked to rate the prompt on a 4-point Likert scale (from strongly disagree to strongly agree): Would [enabler statement] encourage you to adopt/maintain a healthy and sustainable diet? Data were collapsed into a binary variable and % agreement was calculated by combining those who selected ‘agree’ and ‘strongly agree’ to the prompt. ^2^Derived from logistic regression (univariable analysis). ^3^Derived from logistic regression (multivariable analysis) where at least two participant characteristics with *p* < 0.1 in the univariable model were used as covariates. Independent variables that were not associated with the outcome in the univariable models were not included in the multivariable analysis. Multivariable analysis was not conducted for outcomes where there was only one participant characteristic associated. ^4^Qualifications below university level, e.g., secondary school or vocational. Most participants (*n* = 160) were currently enrolled in university. ^5^Includes Mandarin, Cantonese, Vietnamese, Indonesian, Hindi, Malayalam, Gujarati, Bengali, Sinhalese, Tamil, Japanese, German, Korean, Sinhalese. ^6^Plant-based dieters included participants who reported consuming a diet that involved any sort of restriction of animal product intake, i.e., flexitarian, no red meat, pescatarian, vegetarian and vegan. This does not include restriction due to allergies or intolerances. Non plant-based dieters included participants who consumed other diets, i.e., keto, or consumed a non-restricted diet, i.e., omnivore.

### 3.5 Barriers to following a healthy and sustainable diet

**Figure 4:**
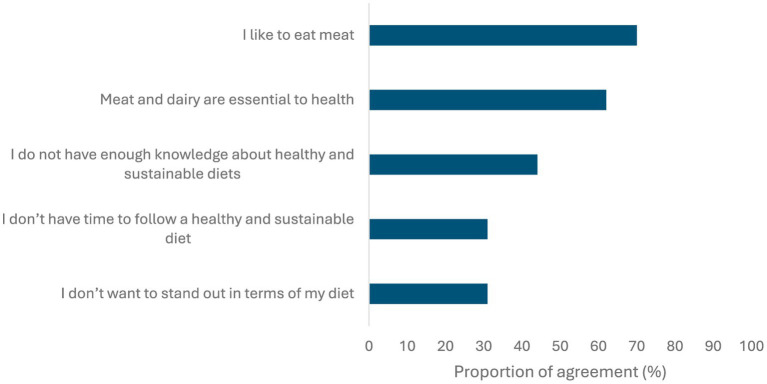
Proportion of participants who agreed to the following barrier statements^†^ in hindering adoption or maintenance of a healthy and sustainable diet (*n* = 238). ^†^Participants were asked to rate their agreement on a 4-point Likert scale (Strongly disagree – strongly agree) of the following barriers in hindering from adopting or maintaining a healthy and sustainable diet. Proportion of agreement was calculated by dividing the number of participants who ‘agreed’ or ‘strongly agreed’ by the total number of participants (*n* = 238).

Figure 4 highlights the proportion of participants (*n* = 238) who selected ‘agree’ or ‘strongly agree’ to the five barrier statements. A majority of participants agreed with the barrier statements ‘I like to eat meat’ (*n* = 166) and ‘meat and dairy are essential to health’ (*n* = 147, 62%).

In the univariable models, participants following a plant-based diet were less likely to agree with the barriers ‘I like to eat meat’ (*p* < 0.001), ‘I do not have enough knowledge on healthy and sustainable diets’ (*p* = 0.009), ‘meat and dairy are essential to health’ (*p* < 0.001), and ‘I do not have time to follow a healthy and sustainable diet’ (*p* = 0.007) compared to those who did not follow a plant-based diet (Table 6). This was also evident in the multivariable models (all *p* < 0.05). Males were more likely to agree with the statements ‘I like to eat meat’ (*p* = 0.018) and ‘I do not have enough knowledge on healthy and sustainable diets’ (*p* = 0.034) compared to females. However, in the multivariable model, only the ‘I like to eat meat’ statement was significantly associated with gender (*p* = 0.016). No other associations were identified between demographic characteristics and barriers to following a healthy and sustainable diet.

**Table 6:** Associations between participant characteristics (*n* = 238) and agreement with barrier statements that showed significant correlations^1^.

Characteristics	I like to eat meat					I do not have enough knowledge on healthy and sustainable diets					Meat and dairy are essential to health					I do not have time to follow a healthy and sustainable diet				
	*n* (%) who agreed to statement	Univariable OR (95% CI)	Univariable *p*-value^2^	Multivariable OR (95% CI)	Multivariable *p*-value^3^	*n* (%) who agreed to statement	Univariable OR (95% CI)	Univariable *p*-value^2^	Multivariable OR (95% CI)	Multivariable *p*-value^3^	*n* (%) who agreed to statement	Univariable OR (95% CI)	Univariable *p*-value^2^	Multivariable OR (95% CI)	Multivariable *p*-value^3^	*n* (%) who agreed to statement	Univariable OR (95% CI)	Univariable *p*-value^2^	Multivariable OR (95% CI)	Multivariable *p*-value^3^
Age		1.0 (0.9, 1.1)	0.810				1.1 (1.0, 1.2)	0.178				1.0 (0.9, 1.1)	0.754				1.0 (0.9, 1.1)	0.968		
Gender			0.035		0.022			0.046		0.147			0.071		0.152			0.662		
Female (reference)	103 (66%)					62 (40%)					93 (60%)					50 (32%)				
Male	58 (82%)	2.3 (1.2, 4.6)	0.018	2.9 (1.2, 7.0)	0.016	39 (55%)	1.8 (1.0, 3.3)	0.034	1.7 (0.9, 3.0)	0.081	50 (70%)	1.6 (0.9, 2.9)	0.119	1.6 (0.8, 3.2)	0.180	23 (32%)	1.0 (0.6, 1.9)	0.959		
Gender diverse or not specified	5 (45%)	0.4 (0.1, 1.5)	0.178	0.4 (0.1, 2.2)	0.309	3 (27%)	0.6 (0.1, 2.2)	0.418	0.6 (0.2, 2.5)	0.513	4 (36%)	0.4 (0.1, 1.4)	0.143	0.4 (0.1, 1.7)	0.227	5 (45%)	1.8 (0.5, 6.1)	0.366		
Education level																				
Below university level^4^ (reference)	129 (70%)					80 (44%)					116 (64%)					62 (34%)				
University qualification	37 (67%)	0.9 (0.5, 1.6)	0.649			24 (44%)	1.0 (0.5, 1.8)	0.992			31 (56%)	0.7 (0.4, 1.4)	0.348			16 (29%)	0.8 (0.4, 1.5)	0.508		
Main language spoken at home																				
English (reference)	119 (69%)					69 (40%)					102 (59%)					53 (31%)				
Other^5^	47 (72%)	1.2 (0.6, 2.2)	0.598			35 (54%)	1.8 (1.0, 3.1)	0.054	1.6 (0.9, 2.9)	0.123	45 (69%)	1.6 (0.9, 2.9)	0.148			25 (38%)	1.4 (0.8, 2.6)	0.253		
Living arrangements																				
Living alone or with partner/housemates (reference)	86 (67%)					53 (41%)					77 (60%)					44 (34%)				
Living with parents and/or other family members	80 (74%)	1.4 (0.8, 2.4)	0.261			51 (47%)	1.3 (0.8, 2.1)	0.377			70 (64%)	1.2 (0.7, 2.1)	0.474			34 (31%)	0.9 (0.5, 1.5)	0.633		
Employment Status			0.658					0.121					0.254					0.692		
Unemployed (reference)	59 (66%)					44 (49%)					61 (69%)					28 (31%)				
Employed part-time/casual	88 (72%)	1.3 (0.7, 2.3)	0.413			46 (37%)	0.6 (0.4, 1.1)	0.081			71 (58%)	0.6 (0.4, 1.1)	0.110			43 (35%)	1.2 (0.7, 2.1)	0.594		
Employed full-time	19 (73%)	1.4 (0.5, 3.6)	0.516			14 (54%)	1.2 (0.5, 2.9)	0.693			15 (58%)	0.6 (0.3, 1.5)	0.306			7 (27%)	0.8 (0.3, 2.1)	0.659		
Student Status			0.292					0.205					0.294					0.057		0.068
Not studying (reference)	26 (63%)					13 (32%)					23 (56%)					9 (22%)				
Studying part-time	9 (60%)	0.9 (0.3, 2.9)	0.815			8 (53%)	2.5 (0.7, 8.2)	0.144			7 (47%)	0.7 (0.2, 2.2)	0.532			2 (13%)	0.5 (0.1, 2.9)	0.477	0.7 (0.1, 3.6)	0.643
Studying full-time	131 (72%)	1.5 (0.7, 3.0)	0.280			83 (46%)	1.8 (0.9, 3.7)	0.107			117 (64%)	1.4 (0.7, 2.8)	0.328			67 (37%)	2.1 (0.9, 4.6)	0.074	2.2 (1.0, 4.9)	0.060
Current diet^6^																				
Non plant-based (reference)	146 (89%)					81 (49%)					128 (78%)					63 (38%)				
Plant-based	20 (27%)	0.0 (0.0, 0.1)	<0.001	0.0 (0.0, 0.1)	<0.001	23 (31%)	0.5 (0.3, 0.8)	0.009	0.5 (0.3, 0.9)	0.014	19 (26%)	0.1 (0.1, 0.2)	<0.001	0.1 (0.1, 0.2)	<0.001	15 (20%)	0.4 (0.2, 0.8)	0.007	0.4 (0.2, 0.8)	0.009

^1^Participants were asked to rate the prompt on a 4-point Likert scale (from strongly disagree to strongly agree): “I like to eat meat.” Data were collapsed into a binary variable and % agreement was calculated by combining those who selected ‘agree’ and ‘strongly agree’ to the prompt. ^2^Derived from logistic regression (univariable analysis). ^3^Derived from logistic regression (multivariable analysis) where at least two participant characteristics with *p* < 0.1 in the univariable model were used as covariates. Independent variables that were not associated with the outcome in the univariable models were not included in the multivariable analysis. Multivariable analysis was not conducted for outcomes where there was only one participant characteristic associated. ^4^Qualifications below university level, e.g., secondary school or vocational. Most participants (*n* = 155) were currently enrolled in university. ^5^Includes Mandarin, Cantonese, Vietnamese, Indonesian, Hindi, Malayalam, Gujarati, Bengali, Sinhalese, Tamil, Japanese, German, Korean, Sinhalese. ^6^Plant-based dieters included participants who reported consuming a diet that involved any sort of restriction of animal product intake, i.e., flexitarian, no red meat, pescatarian, vegetarian and vegan. This does not include restriction due to allergies or intolerances. Non plant-based dieters included participants who consumed other diets, i.e., keto, or consumed a non-restricted diet, i.e., omnivore.

### 3.6 Adherence to healthy and sustainable diets

Table 7 shows the median food group intake and mod-WISH score for each of the 13 food groups that comprise the total mod-WISH score for the participants who completed at least one dietary recall (*n* = 107). Most participants (79%) achieved the maximum score of 10 for added sugar, which indicates full adherence to the recommendation. Conversely, the majority of participants (94%) scored 0 for wholegrains, which indicates intakes below the recommended lower range of 100 g/d.

**Table 7:** Median food group intakes (g/d) and mod-WISH scores of the 13 food groups in the mod-WISH scoring (*n* = 107).

Food groups^1^	Median (IQR) intake (g/d)	mod-WISH recommended intake (lower and upper range) (g/d)	Median (IQR) mod-WISH score	*n* (%) with mod-WISH score of 0	*n* (%) with mod-WISH score of 10
Wholegrains	17.6 (3.7–46.1)	≥125 (100–150)	0 (0–0)	101 (94%)	4 (4%)
Vegetables	161.8 (102.8–231.4)	≥ 300 (200–600)	0 (0–3.1)	74 (69%)	11 (10%)
Fruits	98.1 (43.1–159.3)	≥ 200 (100–300)	0 (0–5.9)	56 (52%)	14 (13%)
Dairy foods	151.4 (82.6–251.6)	250 (0–500)	5.9 (3.2–9.7)	2 (2%)	25 (23%)
Red meat	38.7 (13.3–76.6)	15 (0–30)	0 (0–10)	70 (65%)	27 (25%)
Fish	7.4 (3.6–28.9)	30 (0–100)	2.6 (1.3–10)	16 (15%)	29 (27%)
Eggs	13.9 (4.6–29.2)	15 (0–25)	9.3 (0–10)	35 (33%)	51 (48%)
Chicken and other poultry	47.8 (16.2–85.0)	30 (0–60)	3.5 (0–10)	47 (44%)	41 (38%)
Legumes	27.3 (12.4–55.9)	≥75 (0–100)	3.6 (1.7–7.5)	14 (13%)	15 (14%)
Nuts	5.3 (0–13.3)	50 (0–75)	1.1 (0–2.7)	28 (26%)	2 (2%)
Unsaturated oils	16.8 (11.9–22.6)	40 (20–80)	0 (0–1.3)	71 (66%)	0 (0%)
Saturated oils	13.1 (7.4–21.6)	11.8 (0–11.8)	^2^	59 (55%)	48 (45%)
Added sugars	20.3 (11.7–27.4)	30 (0–30)	^2^	22 (21%)	85 (79%)

^1^Each food group in the mod-WISH score is scored out of 10. A higher score for each of the food groups corresponds to a higher adherence to the food group recommendations for a healthy and sustainable diet (26). ^2^Saturated oils and added sugars were scored on a binary scale, i.e., either 0 or 10.

Out of a total mod-WISH score of 130, the mean score was 49.3 ± 14.5 (range: 21.7–84.4). Higher total mod-WISH score was observed in those following a plant-based diet (54.4 ± 16.5), compared to those on a non plant-based diet (47.1 ± 13.1) (*p* = 0.015) in the univariable models (Table 8). This significant association remained in the multivariable model (*p* = 0.024). Moreover, in the univariable models, participants studying full-time had lower total mod-WISH scores (47.2 ± 15.1), compared to those not studying (54.3 ± 12.0) (*p* = 0.035). In the multivariable model however, this was not significantly associated (global *p* = 0.067).

**Table 8:** Associations between participant characteristics (*n* = 107) and total mod-WISH score.

Characteristics	Total mod-WISH score^1^ Mean (SD)	Univariable Beta (95% CI)	Univariable *p*-value^2^	Multivariable Beta (95% CI)	Multivariable *p*-value^3^
Age		0.4 (−0.6, 1.6)	0.434		
Gender			0.396		
Female (reference)	50.6 (14.6)				
Male	46.7 (13.7)	−4.0 (−10.4, 2.4)	0.222		
Gender diverse or not specified	45.9 (18.4)	−4.8 (−17.0, 7.5)	0.442		
Education level					
Below university level^4^ (reference)	47.9 (14.9)				
University qualification	52.1 (13.7)	4.1 (−1.8, 9.9)	0.171		
Main language spoken at home					
English (reference)	50.1 (14.5)				
Other^5^	47.2 (14.6)	−3.0 (−9.2, 3.2)	0.346		
Living arrangements					
Living alone or with partner/housemates (reference)	51.0 (13.6)				
Living with parents and/or other family members	47.9 (15.2)	3.1 (−2.5, 8.7)	0.278		
Employment status			0.118		
Unemployed (reference)	45.3 (13.7)				
Employed part-time/casual	51.1 (15.9)	5.8 (−0.3, 12.0)	0.063		
Employed full-time	52.2 (10.1)	7.0 (−1.4, 15.4)	0.103		
Student status			0.033		0.067
Not studying (reference)	54.3 (12.0)				
Studying part-time	59.0 (7.3)	4.8 (−9.1, 18.6)	0.495	3.0 (−10.8, 16.8)	0.667
Studying full-time	47.2 (15.1)	−7.1 (−13.7, −0.5)	0.035	−6.5 (−13.0, −0.0)	0.048
Current diet^6^					
Non plant-based (reference)	47.1 (13.1)				
Plant-based	54.4 (16.5)	7.3 (1.4, 13.2)	0.015	6.8 (0.9, 12.7)	0.024

^1^The mod-WISH score is based on the World Index for Sustainability and Health (26), which measures adherence to a healthy and sustainable diet. Mod-WISH takes into account the 2025 EAT-Lancet Planetary Health Diet guidelines (28). Possible score range is 0–130. ^2^Derived from linear regression (univariable analysis). ^3^Derived from linear regression (multivariable analysis) where student status and current diet were used as covariates. Independent variables that were not associated with the outcome in the univariable models were not included in the multivariable analysis. ^4^Qualifications below university level, e.g., secondary school or vocational. Most participants (*n* = 66) were currently enrolled in university. ^5^Includes Mandarin, Cantonese, Vietnamese, Indonesian, Hindi, Malayalam, Gujarati, Bengali, Sinhalese, Tamil, Japanese, German, Korean, Sinhalese. ^6^Plant-based dieters included participants who reported consuming a diet that involved any sort of restriction of animal product intake, i.e., flexitarian, no red meat, pescatarian, vegetarian and vegan. This does not include restriction due to allergies or intolerances. Non plant-based dieters included participants who consumed a non-restricted diet, i.e., omnivore, or consumed other diets, i.e., keto.

## 4 Discussion

### 4.1 Summary of results

This study examined the adherence and factors influencing adoption of healthy and sustainable diets among young Australian adults, and examined associations between these outcomes with demographic and dietary characteristics.

Although most participants demonstrated sound general nutrition knowledge, comprehension of healthy and sustainable diet composition was limited. When adjusting for covariates, healthy and sustainable dietary knowledge was higher among those on a plant-based diet. Furthermore, the majority of participants did not perceive healthy and sustainable diets as convenient, accessible or affordable. Those on a plant-based diet were more likely to perceive them as affordable and palatable. Conversely, full-time employed individuals were less likely to think these diets were convenient. Preference for meat and dairy products and perceived health benefits emerged as primary barriers to adopting these diets. Males reported having stronger preferences for meat, compared to females. Identified enablers included awareness of environmental and animal welfare benefits, which were particularly salient for females and those on a plant-based diet. Having accessible and affordable food options were valued more by employed individuals.

Overall, adherence to healthy and sustainable diets among young Australian adults remained suboptimal. Individuals on a plant-based diet had significantly higher mod-WISH scores compared to those on an unrestricted diet.

### 4.2 Adherence to healthy and sustainable diets

The low adherence to healthy and sustainable diets among young adults identified in this study is substantiated by global evidence (29, 30). For example, a study using the WISH tool in Mexico, United States and Canada found that young adults (aged 20–39 years old) had significantly lower scores with a difference of up to 5%, compared to older age groups (30). This raises health concerns for young adults whilst furthering negative environmental impacts, which young adults are motivated to take action on (7), highlighting a gap between intention and behavior. Low adherence to healthy and sustainable diets can negatively impact health status, and increase risk of chronic conditions (31–34), which young Australians are already at risk at (10, 35). Additionally, low adherence is associated with higher greenhouse gas emissions and land use (36). Considering that dietary patterns and plant-based diet quality tracks from adolescence into young adulthood to older adulthood (37, 38), it is imperative to build healthy and sustainable dietary habits that align with young adults’ social values, to maintain good health and environmental sustainability in the long term.

In the current study, full-time students had lower adherence to healthy and sustainable diets in the univariable model. Although the multivariable model found no significant differences, the model suggested an effect that approached significance. Overall, adherence was low, and given that the majority of the sample was full-time students, this group remains a priority group for dietary intervention. Previous evidence indicates university students have poorer diet quality, compared to the general population (39). The transition from secondary school to university, where students may often experience several academic, financial, social and personal challenges (40), can lead to food insecurity and poor mental health (9, 39, 41). These factors negatively impact food behavior and diet quality. As such, a focus on preventative nutrition efforts may be needed in young adults, particularly full-time tertiary students, by promoting intakes toward healthy and sustainable dietary recommendations (1).

With the exception of added sugars, none of the mod-WISH food group recommendations were met by most participants. No participants met the recommendations for unsaturated oils, while nuts, wholegrains, vegetables, fruits, legumes, fish and dairy were under-consumed. On the other hand, red meat, poultry and eggs were over-consumed compared to recommendations. Although fish and dairy have a high environmental impact (42), a moderate amount is recommended due to their protective health benefits and key micronutrient profiles (1). As such, dietary interventions for young adults should promote healthy plant-source foods, while also supporting moderate dairy and fish consumption to meet nutrient requirements, e.g., calcium and omega-3 s (43). For a higher positive impact on health and the environment, a focus on increasing legume and nut intakes may reduce the overconsumption of red meat, poultry, and eggs. This is because legumes and nuts are regarded as traditional substitutes for meat due to their similar nutritional profiles (44). To encourage consumption toward healthy and sustainable dietary patterns, an exploration of the factors influencing adoption, such as knowledge and attitudes, can identify opportunities for behavior change.

### 4.3 Knowledge of, and attitudes and barriers toward healthy and sustainable diets

Nutrition knowledge scores from the current study indicate that the sample was somewhat informed about general nutrition concepts. However, key knowledge gaps regarding healthy and sustainable dietary guidelines were identified. Participants were not aware that animal-source foods need to be moderated in a healthy and sustainable dietary pattern. Despite this, most participants reported that they had enough knowledge about healthy and sustainable diets. This aligns with previous evidence where young adults in four European countries demonstrated low awareness and objective knowledge of the composition of plant-based diets (23). This raises a concern as knowledge of dietary guidelines and knowledge of general nutrition concepts are positively associated with adherence to dietary recommendations (45–47). As such, healthy and sustainable dietary interventions for young adults need to focus on improving knowledge and awareness of what food groups to moderate and focus on.

Although the sample generally had favorable attitudes toward healthy and sustainable diets, most did not believe that these diets were convenient, affordable or accessible. Additionally, when investigating enablers, many participants reported that having more access to healthy and sustainable food options that are affordable would enable them to follow these diets. A scoping review (48) and a previous qualitative study in young Australian adults (15) also revealed that cost and lack of food options were barriers to following a healthy and sustainable diet. Convenience and affordability are extrinsic factors that significantly drive food choice, and can hinder individuals from adopting healthy and sustainable diets (49). Although evidence shows that educational nutrition interventions have the potential to motivate individuals toward healthy eating (50, 51), future interventions need to also consider intervention functions (52) beyond education, such as environmental restructuring, skills training and enablement, to overcome external factors such as cost and accessibility.

Participants reported several other barriers that may hinder their adoption toward healthy and sustainable diets. Most agreed that their preference for consuming meat, and belief that meat and dairy were essential for health, were both factors that hindered them from adopting healthy and sustainable diets. These findings align with the Australian Dietary Guidelines where meat and dairy are considered core food groups (43). Additionally, meat consumption remains dominant in Australian diets (53). Given the role of meat and dairy within the context of healthy Australian diets and culture, an emphasis on substituting discretionary foods for healthy plant-based alternatives, may help to improve adherence to healthy and sustainable diets in this age group, while also maintaining nutrient adequacy.

### 4.4 Demographic groups who need targeted intervention

Interestingly, findings indicate that young adults who were employed were less likely to perceive healthy and sustainable diets as convenient to follow, compared to those who were unemployed. Furthermore, they were more likely to agree that improving access to cheaper healthy and sustainable foods may encourage them toward healthy and sustainable diets. As such, individual-level opportunities to overcome convenience and accessibility concerns of healthy and sustainable foods, e.g., practical strategies on simple meals and snacks (54), may aid in changing their attitudes and ultimately, their behaviors (55). Moreover, previous studies report several barriers among employed young adults hinder them from following healthy diets. For young adults, the transition from study to employment further impacts mental health due to a number of psychosocial job stressors (56). These include low job security, low job control and high work demands. Long work hours, which are associated with perceived time barriers, may also have adverse impacts on healthy eating among young adults (57). Perceived time constraints have consistently been reported as a barrier to healthy eating (48), and is associated with unhealthy dietary practices (58). For example, time spent working and commuting is associated with increased purchases of take-away foods which in turn reduces consumption of healthy foods (59). Contrasting to the literature, most participants in the current study did not report lack of time as a barrier to following a healthy and sustainable diet. This may be due to recruiting a homogenous sample with the majority being full-time students and a higher unemployment rate compared to the national average (60).

Another group of young adults that may require more tailored approaches to behavior change are males. Males in the current study were more likely to agree that they liked eating meat compared to females. Historically, meat consumption has been considered as a sign of masculinity (61), which may consequently lead males to believe that plant-based diets are emasculating (62). This poses a significant barrier to following a healthy and sustainable diet in this group. They were also less likely to believe that understanding the animal welfare benefits encouraged them to follow healthy and sustainable diets. As such, focusing on other motivators such as health and fitness benefits, rather than the animal benefits, may effectively target men’s behavior change. Overall, future interventions need to consider the unique challenges different groups of young adults encounter, for effective and tailored behavior change.

### 4.5 Strengths and limitations

A strength of the study was its collection and analysis of both survey and dietary data using validated methods to identify associations between knowledge, attitudes, level of adherence to healthy and sustainable diets and demographic and dietary characteristics. The ASA24-Australia tool is an online, validated tool designed to reduce misreporting from interviewer bias and social acceptability bias.

There were some limitations that may affect the generalizability of the results. Firstly, although the study was sufficiently powered to detect differences in dietary guideline knowledge scores between diet types (i.e., omnivore vs. plant-based), the variability of the sample was likely not sufficient in detecting differences in other correlates, e.g., attitudes, enablers and barriers, between demographic groups. This was indicated by large confidence interval ranges when performing analyses. Secondly, a convenience sampling strategy may have introduced sampling bias, leading to a sample that was not nationally representative of the young Australian adult population. The sample recruited were mostly women, educated, and current tertiary students. Thirdly, 24-h recalls are prone to recall bias, and the 2011-13 AUSNUT databases may not include all novel plant-based food items. These limitations may ultimately lead to misreporting of some foods.

## 5 Conclusion

Results from the study further underscores the need to target young adults, including tertiary students, for preventative interventions that promote healthy and sustainable dietary patterns. These interventions should involve the promotion of nuts, wholegrains, vegetables, fruits and legumes to substantially improve young adults’ adherence to a healthy and sustainable diet. Interventions for this age group need to consider and act on the unique challenges young Australian adults encounter, especially as they emerge into work or further study, when practising healthier and more sustainable diets. Findings from this study can be used to initiate the planning and development of content for a healthy and sustainable nutrition intervention tailored toward young adults.

## Data Availability

The datasets presented in this article are not readily available because data from this research project are unable to be shared as participants did not consent to the sharing of their data. Requests to access the datasets should be directed to BTL, s223212335@deakin.edu.au.

## Glossary

Adherence: The degree to which an individual’s food intake aligns with the recommendations of a specific dietary pattern
Healthy and sustainable diets: Dietary patterns that promote health and wellbeing, while minimizing negative environmental and social impacts
