# Supplementary material for: Adherence to and factors associated with the adoption of healthy and sustainable diets in young Australian adults: a cross-sectional study
Source: Front Nutr. 2026 Jul 22;13:1862059. doi: 10.3389/fnut.2026.1862059 (PMC13463707; doi:10.3389/fnut.2026.1862059)
Supplement: Supplementary file 2 [file Table_2.docx]

## Supplementary Material 2. Description of food components, food inclusions and food exclusions from the World Index of Sustainability and Health dietary index tool (26)

| Food group | WISH definition | | Inclusions | Exclusions | |  |  |
| --- | --- | --- | --- | --- | --- | --- | --- |
| Wholegrains | The intake of this food group can be defined as the average daily consumption of whole grains such as rice, wheat and corn (bran, germ, and endosperm in their natural proportion) or whole grain products in the form of breakfast cereals, bread, pasta, biscuits, muffins, tortillas, pancakes, and other sources. | | Whole grains (i.e. with bran germ and endosperm in their natural proportions) e.g. brown rice, rolled oats. Grain products made predominantly (>75%) with wholemeal/ wholewheat flour/ oatmeal e.g. wholemeal bread, wholewheat breakfast cereals.  Whole grains in grain products e.g. rolled oats in porridge | Refined grains where the bran, germ and/ or endosperm have been removed during processing e.g. white rice. Isolated bran or germ products e.g. wheat bran. | |  |  |
| Vegetables | This food group is defined by EAT-Lancet as fresh, frozen, cooked, canned, or dried vegetables, and excludes legumes and salted or pickled vegetables, juices, nuts, and seeds, and starchy vegetables such as potatoes or corn. | | Any fresh, frozen, cooked, canned or dried vegetables | Any starchy vegetable including potatoes, sweet potatoes, corn, pumpkin and squash Pickled or salted vegetables Vegetable juices Any vegetable-based chips or fries Vegetables used in condiments | |  |  |
| Fruit | The food group is defined as fruits, which are fresh, frozen, cooked, canned, or dried but excluding fruit juices, salted or pickled fruits and nuts or seeds. | | Any fresh, frozen, cooked, canned or dried fruit | Fruit juice and fruit syrups, including those from canned fruit Salted or pickled fruits Ultra-processed fruit foods e.g. banana chips Fruit used in condiments e.g. jam | |  |  |
| Dairy foods | The definition of dairy foods includes whole or skimmed milk or derivative equivalents (e.g., cheese, yoghurt or curd), excluding butter and cream. | | Milk (fluid, or fluid prepared from powder) from cow, sheep or goat Yoghurt  Cheese Custard Dairy alternatives of above e.g. soy, almond, with added calcium | Butter and cream Milk solids or powder in mixed foods Whey products | |  |  |
| Red meat | Red meat is beef, pork, lamb, and goat including red meat in its processed form but excluding poultry, fish, eggs. | | Beef, pork, lamb, goat, kangaroo, crocodile meat and organ meat Processed or ultra-processed meats e.g. beef mince, sausages | Meat used in condiments e.g. paste, pate | |  |  |
| Fish | This food group includes fish and shellfish such as mussels and shrimp but no distinction for fish rich in omega-3 was made. | | Any fresh or canned fish or other seafood  Fish eggs (caviar) | Fish used in condiments e.g. paste and sauce | |  |  |
| Eggs | This food group includes eggs from chickens and ducks, but excludes fish eggs. | | Chicken and duck eggs Egg powder | Eggs used in condiments e.g. mayonnaise | |  |  |
| Chicken and other poultry | This food group includes meat from chicken, ducks, geese etc. | | Chicken, duck, turkey, and other poultry meat or organ meat Processed or ultra-processed chicken/ poultry e.g. chicken mince and chicken sausages | Chicken/ poultry used in condiments e.g. pate | |  |  |
| Legumes | This food group includes fresh, frozen, cooked, canned, or dried legumes, beans and lentils, but also includes soy foods and peas. | | Beans, legumes, lentils, soy foods e.g. tofu and tempeh, peas Bean sprouts, alfalfa sprouts | Legumes used in condiments e.g. black bean sauce | |  |  |
| Nuts | This food group includes tree nuts and ground nuts (including peanuts). | | All nuts e.g. peanuts, walnuts, almonds Nut butters e.g. peanut butter | All seeds Nuts used in condiments e.g. satay sauce | |  |  |
| Unsaturated oils | Examples of unsaturated oils are olive, soybean, rapeseed, sunflower and peanut oil. | | Plant-based oils e.g. sunflower, canola, olive Margarine | Palm oil Coconut (copha) oil Shortening and other hydrogenated oils | |  |  |
| Saturated oils | Saturated oils originate from oils like palm oil, and from dairy fats, lard or tallows. | | Palm oil and coconut (copha) oil Any animal-based oil e.g. lard, suet Shortening and other hydrogenated oils Butter and cream | Fats from the 'Dairy Foods' group | |  |  |
| Added sugars | Added sugars include measured added sugar and sugar sweetened beverages | | Any type of sugar (e.g. brown, white, granulated, caster, icing) added into foods or beverages  Sugar-based sweeteners e.g. honey | Artificial sweeteners e.g. aspartame | |  |  |
|  | |  | | |  | |  |
