# Supplementary material for: Adherence to and factors associated with the adoption of healthy and sustainable diets in young Australian adults: a cross-sectional study
Source: Front Nutr. 2026 Jul 22;13:1862059. doi: 10.3389/fnut.2026.1862059 (PMC13463707; doi:10.3389/fnut.2026.1862059)
Supplement: Supplementary file 3 [file Table_3.docx]

## Supplementary Material 3. Mean total energy and macronutrient intakes among participants who provided dietary data (n=107) by gender.

| **Intake** | **Gender (n)** | **Mean (SD)** |
| --- | --- | --- |
| Total Energy (kJ/d) | Total (n=107) | 7260 (1989) |
|  | Female (n=73) | 7161 (1765) |
|  | Male (n=28) | 7863 (2363) |
|  | Gender diverse or not specified (n=6) | 5649 (1877) |
| Protein (E%) | Total (n=107) | 19 (4) |
|  | Female (n=73) | 19 (4) |
|  | Male (n=28) | 21 (6) |
|  | Gender diverse or not specified (n=6) | 20 (7) |
| Fat (E%) | Total (n=107) | 36 (5) |
|  | Female (n=73) | 37 (4) |
|  | Male (n=28) | 35 (6) |
|  | Gender diverse or not specified (n=6) | 32 (4) |
| Carbohydrates (E%) | Total (n=107) | 42 (6) |
|  | Female (n=73) | 42 (5) |
|  | Male (n=28) | 42 (7) |
|  | Gender diverse or not specified (n=6) | 45 (10) |
