# Supplementary material for: Adherence to and factors associated with the adoption of healthy and sustainable diets in young Australian adults: a cross-sectional study
Source: Front Nutr. 2026 Jul 22;13:1862059. doi: 10.3389/fnut.2026.1862059 (PMC13463707; doi:10.3389/fnut.2026.1862059)
Supplement: Supplementary file 4 [file Table_4.docx]

## Supplementary Material 4. Proportion of participants answering each general nutrition knowledge question (n=296)

| Question† | n selected ‘true’ (%) | n selected ‘false’ (%) | n selected ‘don’t know’ (%) |
| --- | --- | --- | --- |
| Lentils contain only a few useful nutrients, therefore their health benefit is not great.  *[Correct answer: false]* | 26 (9%) | 237 (80%)* | 34 (11%) |
| If you have eaten high-fat foods, you can reverse the effects by eating apples.  *[Correct answer: false]* | 22 (7%) | 237 (80%)* | 38 (13%) |
| If cream is whipped it contains less calories than in its liquid form.  *[Correct answer: false]* | 28 (9%) | 215 (72%)* | 54 (18%) |
| A healthy meal should consist of half meat, a quarter vegetables and a quarter grains.  *[Correct answer: false]* | 98 (33%) | 170 (57%)* | 29 (10%) |
| Fat contains fewer calories than the same amount of fibre.  *[Correct answer: false]* | 25 (8%) | 200 (67%)* | 72 (24%) |
| A salad dressing made with mayonnaise is as healthy as the same dressing made with mustard.  *[Correct answer: false]* | 41 (14%) | 195 (66%)* | 61 (21%) |
| Fat is always bad for your health; you should therefore avoid it as much as possible.  *[Correct answer: false]* | 18 (6%) | 275 (93%)* | 4 (1%) |
| Pasta with tomato sauce is healthier than pasta with cream sauce.  *[Correct answer: true]* | 178 (60%)* | 59 (20%) | 605 (20%) |
| A balanced diet implies eating all foods in the same amounts.  *[Correct answer: false]* | 25 (8%) | 264 (89%)* | 8 (3%) |
| The health benefit of fruit and vegetables lies alone in the supply of vitamins and minerals.  *[Correct answer: false]* | 66 (22%) | 208 (70%)* | 23 (8%) |
| Bacon contains more calories than ham.  *[Correct answer: true]* | 140 (47%)* | 29 (10%) | 128 (43%) |
| Oily fish (salmon, tuna) contain healthier fats than red meat.  *[Correct answer: true]* | 215 (72%)* | 21 (7%) | 61 (21%) |
| To eat healthily, you should eat less fat. Whether you also eat more fruit and vegetables does not matter.  *[Correct answer: false]* | 41 (14%) | 238 (80%)* | 18 (6%) |
| A scoop of chocolate ice cream is just as healthy as a scoop of lemon sorbet.  *[Correct answer: false]* | 54 (18%) | 187 (63%)* | 56 (19%) |
| The same amount of beef steak and chicken breast contains equally many calories.  *[Correct answer: false]* | 17 (6%) | 229 (77%)* | 51 (17%) |
| The same amount of sugar and fat contains equally many calories.  *[Correct answer: false]* | 27 (9%) | 205 (69%)* | 65 (22%) |
| A sandwich with mozzarella contains as many calories as the same sandwich with cheddar cheese.  *[Correct answer: false]* | 47 (16%) | 146 (49%)* | 104 (35%) |
| For a healthy diet, dairy products should be consumed in the same amounts as fruit and vegetables.  *[Correct answer: false]* | 25 (8%) | 226 (79%)* | 46 (15%) |
| Skim milk contains fewer minerals than full-fat milk.  *[Correct answer: false]* | 101 (34%) | 88 (30%)* | 108 (36%) |
| Brown sugar is much healthier than white sugar.  *[Correct answer: false]* | 118 (40%) | 125 (42%)* | 54 (18%) |

†Questions were from a validated 20-item nutrition knowledge questionnaire (22)
*Correct answer
