# Supplementary material for: Adherence to and factors associated with the adoption of healthy and sustainable diets in young Australian adults: a cross-sectional study
Source: Front Nutr. 2026 Jul 22;13:1862059. doi: 10.3389/fnut.2026.1862059 (PMC13463707; doi:10.3389/fnut.2026.1862059)
Supplement: Supplementary file 5 [file Table_5.docx]

## Supplementary Material 5. Proportion of participants (n=276) answering each healthy and sustainable diet knowledge question

| Food groups† | n selected ‘encouraged’ (%) | n selected ‘optional’ (%) | n selected ‘discouraged’ (%) | n selected ‘don’t know’ (%) |
| --- | --- | --- | --- | --- |
| Wholegrains  *[Correct answer: encouraged]* | 253 (92%)* | 16 (6%) | 3 (1%) | 4 (1%) |
| Refined grains  *[Correct answer: discouraged]* | 52 (19%) | 92 (33%) | 94 (24%)* | 38 (14%) |
| Vegetables  *[Correct answer: encouraged]* | 270 (98%)* | 3 (1%) | 1 (<1%) | 2 (1%) |
| Fruits  *[Correct answer: encouraged]* | 254 (92%)* | 19 (7%) | 0 (0%) | 3 (1%) |
| Red Meat  *[Correct answer: discouraged]* | 93 (34%) | 117 (42%) | 56 (20%)* | 10 (4%) |
| Poultry  *[Correct answer: optional]* | 136 (49%) | 121 (44%)* | 9 (3%) | 10 (4%) |
| Fish  *[Correct answer: optional]* | 187 (68%) | 75 (27%)* | 8 (3%) | 6 (2%) |
| Dairy  *[Correct answer: optional]* | 144 (52%) | 115 (42%)* | 13 (5%) | 4 (1%) |
| Junk foods  *[Correct answer: discouraged]* | 4 (1%) | 23 (8%) | 245 (89%)* | 4 (1%) |
| Eggs  *[Correct answer: optional]* | 185 (67%) | 81 (29%)* | 4 (1%) | 6 (2%) |
| Legumes  *[Correct answer: encouraged]* | 217 (79%)* | 36 (13%) | 4 (1%) | 19 (7%) |
| Soy foods  *[Correct answer: encouraged]* | 101 (37%)* | 131 (47%) | 16 (6%) | 28 (10%) |
| Nuts and seeds  *[Correct answer: encouraged]* | 217 (79%)* | 50 (18%) | 3 (1%) | 6 (2%) |

†Participants were asked to select if the following food groups are encouraged, optional or discouraged in a healthy and sustainable diet. Correct answers are based on the EAT-Lancet Planetary Health Diet guidelines.(1)
*Correct answer
